# Supplementary material for: Multi-Class Determination of 64 Illicit Compounds in Dietary Supplements Using Liquid Chromatography–Tandem Mass Spectrometry
Source: Molecules. 2020 Sep 24;25(19):4399. doi: 10.3390/molecules25194399 (PMC7583945; doi:10.3390/molecules25194399)
Supplement: Supplementary file 1 [file molecules-25-04399-s001.pdf]

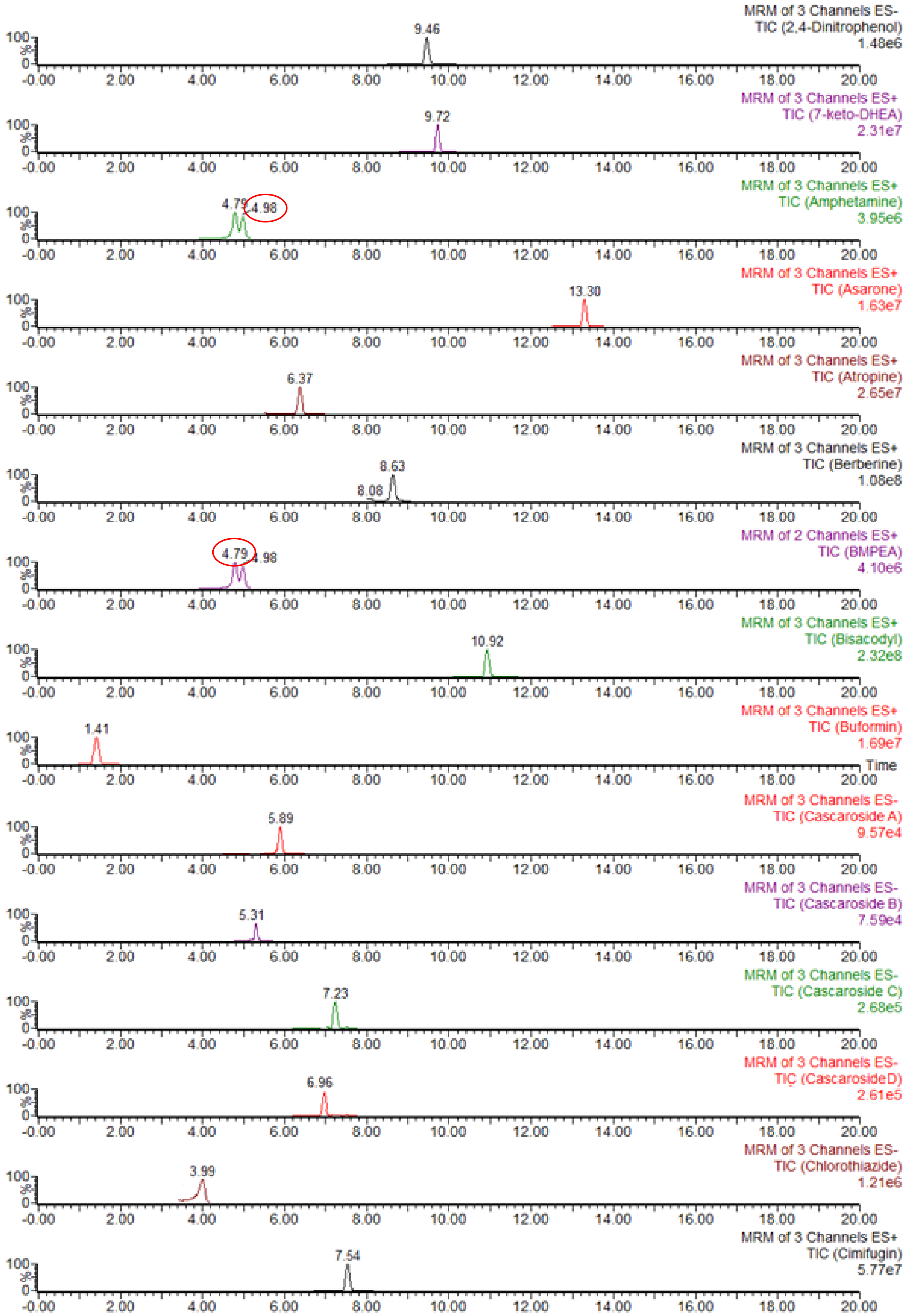
MRM of 3 Channels ES-
TIC (2,4-Dinitrophenol)
1.48e6
9.46
MRM of 3 Channels ES+
TIC (7-keto-DHEA)
2.31e7
9.72
MRM of 3 Channels ES+
TIC (Amphetamine)
3.95e6
4.79
4.98
MRM of 3 Channels ES+
TIC (Asarone)
1.63e7
13.30
MRM of 3 Channels ES+
TIC (Atropine)
2.65e7
6.37
MRM of 3 Channels ES+
TIC (Berberine)
1.08e8
8.63
8.08
MRM of 2 Channels ES+
TIC (BMPEA)
4.10e6
4.79
4.98
MRM of 3 Channels ES+
TIC (Bisacodyl)
2.32e8
10.92
MRM of 3 Channels ES+
TIC (Buformin)
1.69e7
1.41
Time
MRM of 3 Channels ES-
TIC (Cascaroside A)
9.57e4
5.89
MRM of 3 Channels ES-
TIC (Cascaroside B)
7.59e4
5.31
MRM of 3 Channels ES-
TIC (Cascaroside C)
2.68e5
7.23
MRM of 3 Channels ES-
TIC (Cascaroside D)
2.61e5
6.96
MRM of 3 Channels ES-
TIC (Chlorothiazide)
1.21e6
3.99
MRM of 3 Channels ES+
TIC (Cimifugin)
5.77e7
7.54
100
%
0
-0.00
2.00
4.00
6.00
8.00
10.00
12.00
14.00
16.00
18.00
20.00

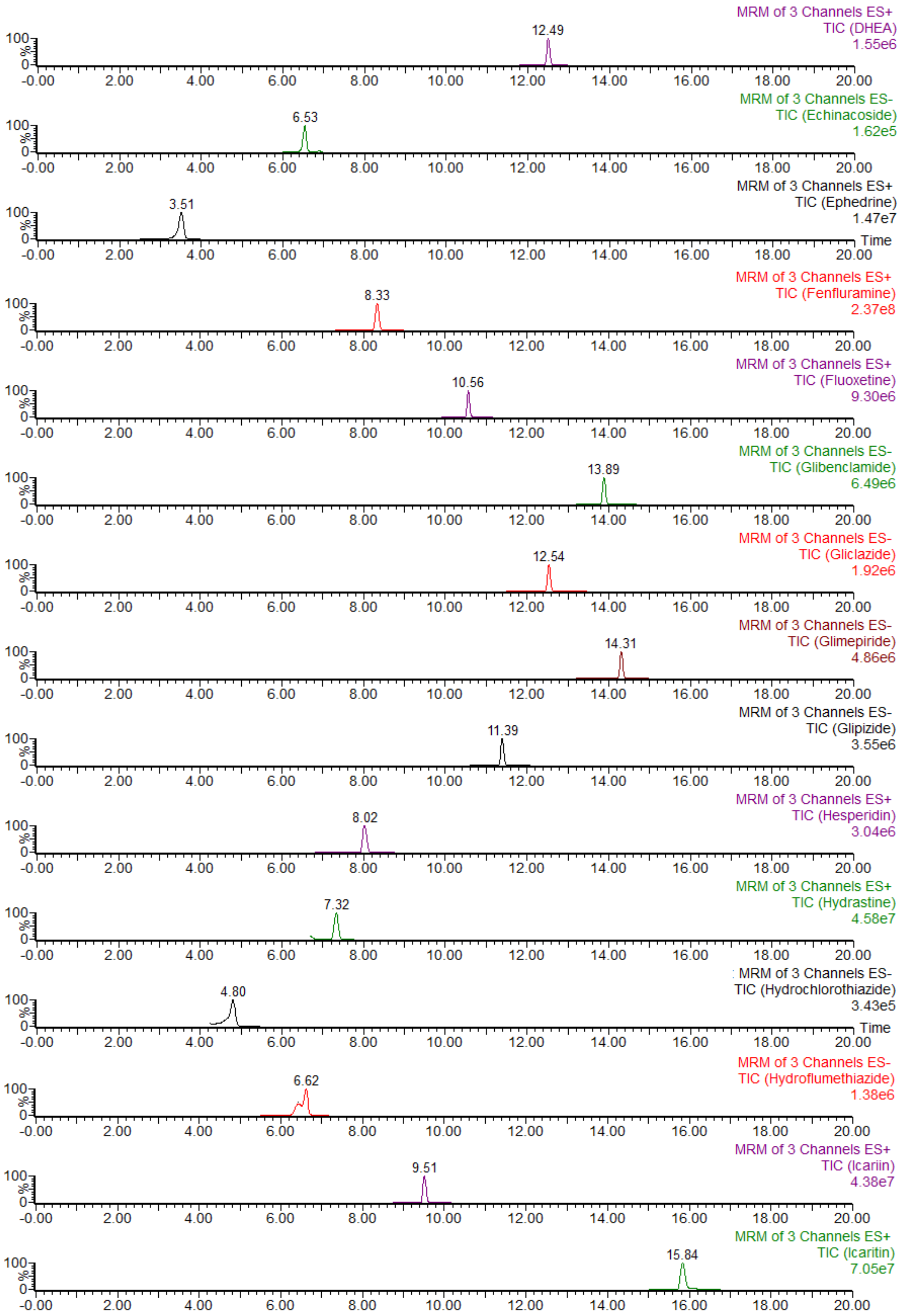
MRM of 3 Channels ES+
TIC (DHEA)
1.55e6
12.49
MRM of 3 Channels ES-
TIC (Echinacoside)
1.62e5
6.53
MRM of 3 Channels ES+
TIC (Ephedrine)
1.47e7
3.51
MRM of 3 Channels ES+
TIC (Fenfluramine)
2.37e8
8.33
MRM of 3 Channels ES+
TIC (Fluoxetine)
9.30e6
10.56
MRM of 3 Channels ES-
TIC (Glibenclamide)
6.49e6
13.89
MRM of 3 Channels ES-
TIC (Gliclazide)
1.92e6
12.54
MRM of 3 Channels ES-
TIC (Glimepiride)
4.86e6
14.31
MRM of 3 Channels ES-
TIC (Glipizide)
3.55e6
11.39
MRM of 3 Channels ES+
TIC (Hesperidin)
3.04e6
8.02
MRM of 3 Channels ES+
TIC (Hydrastine)
4.58e7
7.32
MRM of 3 Channels ES-
TIC (Hydrochlorothiazide)
3.43e5
4.80
MRM of 3 Channels ES-
TIC (Hydroflumethiazide)
1.38e6
6.62
MRM of 3 Channels ES+
TIC (Icariin)
4.38e7
9.51
MRM of 3 Channels ES+
TIC (Icaritin)
7.05e7
15.84
100
%
0
Time
-0.00
2.00
4.00
6.00
8.00
10.00
12.00
14.00
16.00
18.00
20.00

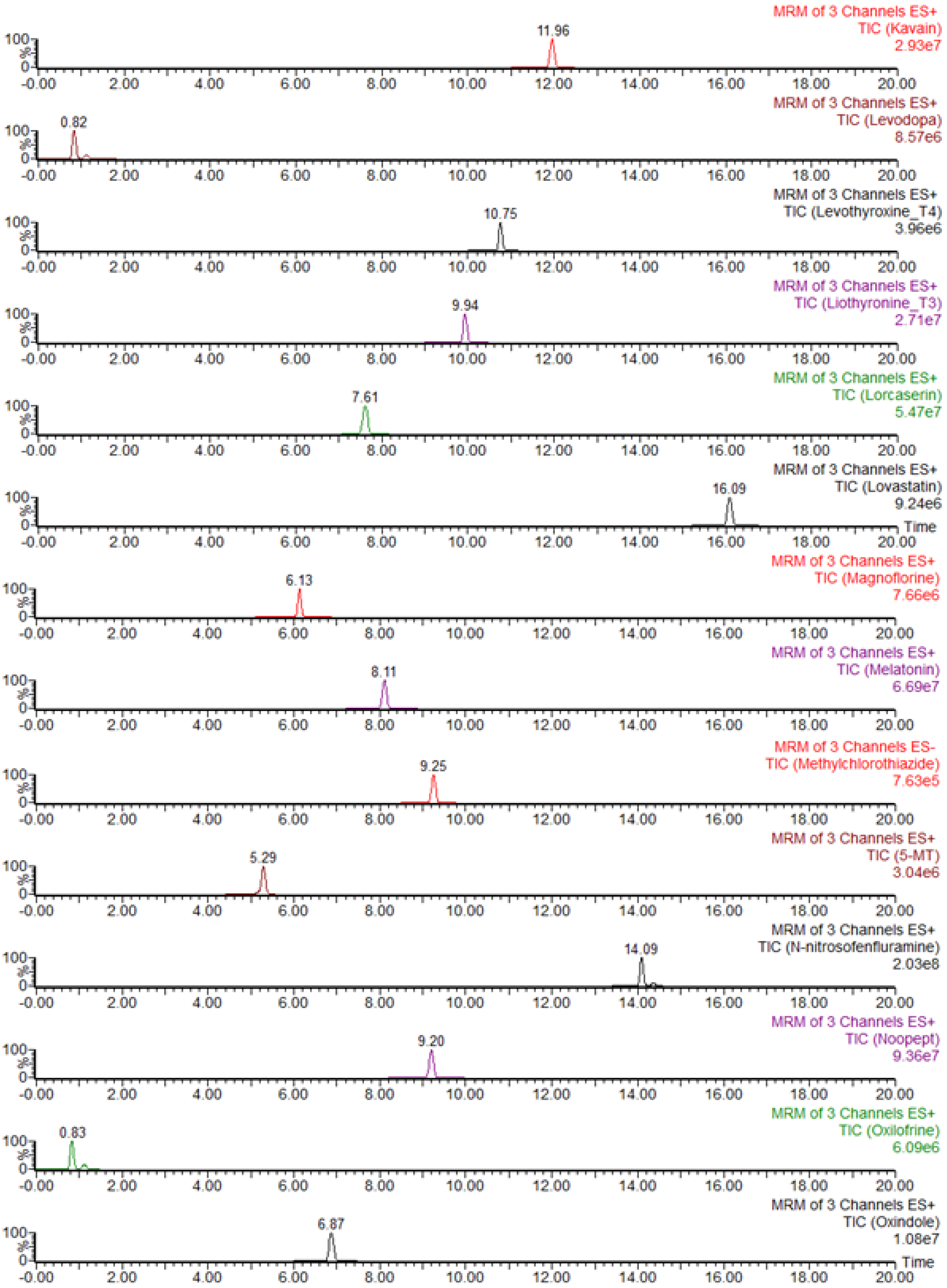

MRM of 3 Channels ES+
TIC (Kavain)
2.93e7
11.96
MRM of 3 Channels ES+
TIC (Levodopa)
8.57e6
0.82
MRM of 3 Channels ES+
TIC (Levothyroxine_T4)
3.96e6
10.75
MRM of 3 Channels ES+
TIC (Liothyronine_T3)
2.71e7
9.94
MRM of 3 Channels ES+
TIC (Lorcaserin)
5.47e7
7.61
MRM of 3 Channels ES+
TIC (Lovastatin)
9.24e6
16.09
Time
MRM of 3 Channels ES+
TIC (Magnoflorine)
7.66e6
6.13
MRM of 3 Channels ES+
TIC (Melatonin)
6.69e7
8.11
MRM of 3 Channels ES-
TIC (Methylchlorothiazide)
7.63e5
9.25
MRM of 3 Channels ES+
TIC (5-MT)
3.04e6
5.29
MRM of 3 Channels ES+
TIC (N-nitrosofenfluramine)
2.03e8
14.09
MRM of 3 Channels ES+
TIC (Noopept)
9.36e7
9.20
MRM of 3 Channels ES+
TIC (Oxilofrine)
6.09e6
0.83
MRM of 3 Channels ES+
TIC (Oxindole)
1.08e7
6.87
Time
100
%
0
-0.00 2.00 4.00 6.00 8.00 10.00 12.00 14.00 16.00 18.00 20.00

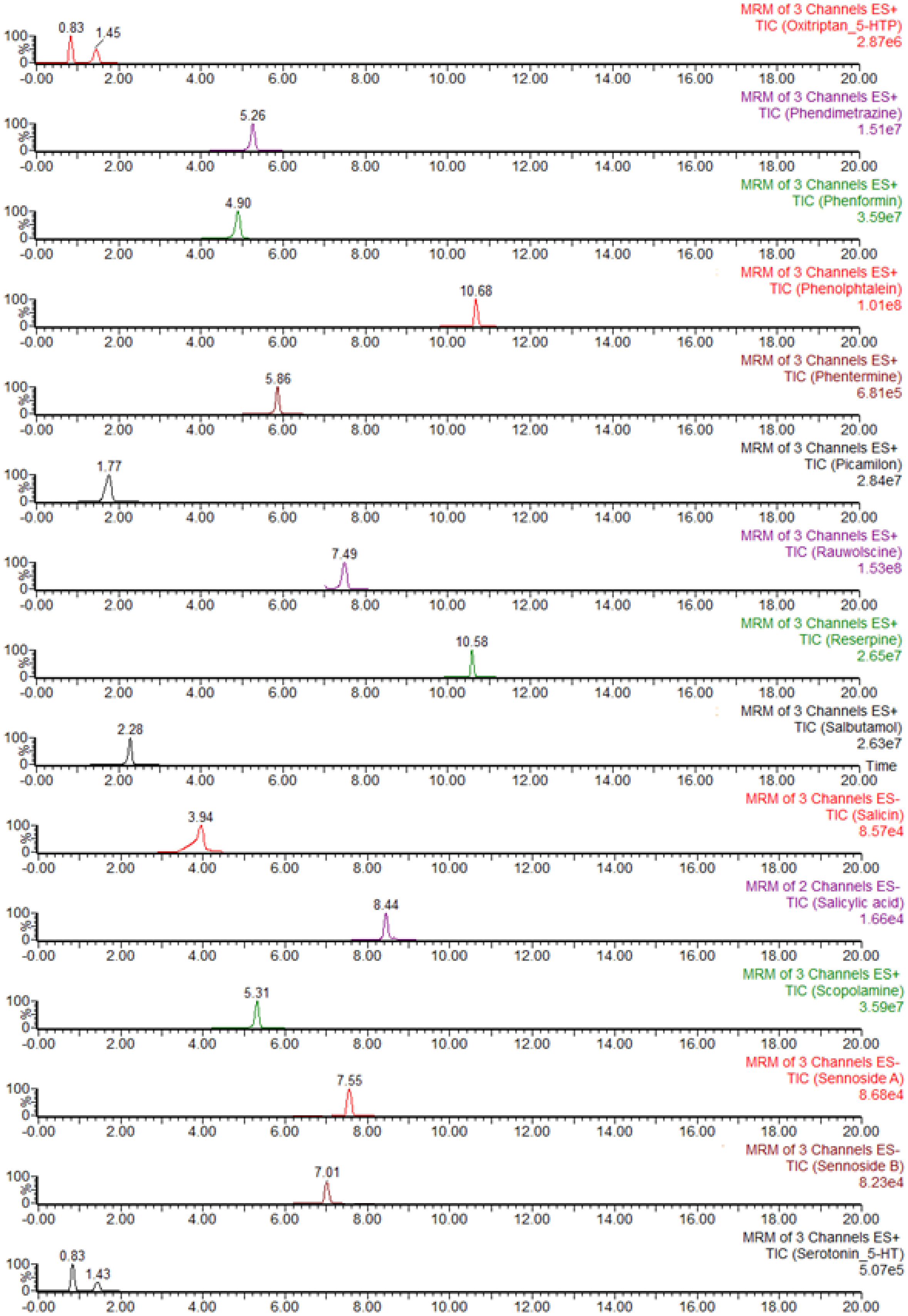
MRM of 3 Channels ES+
TIC (Oxitriptan_5-HTP)
2.87e6
0.83
1.45
MRM of 3 Channels ES+
TIC (Phendimetrazine)
1.51e7
5.26
MRM of 3 Channels ES+
TIC (Phenformin)
3.59e7
4.90
MRM of 3 Channels ES+
TIC (Phenolphtalein)
1.01e8
10.68
MRM of 3 Channels ES+
TIC (Phentermine)
6.81e5
5.86
MRM of 3 Channels ES+
TIC (Picamilon)
2.84e7
1.77
MRM of 3 Channels ES+
TIC (Rauwolscine)
1.53e8
7.49
MRM of 3 Channels ES+
TIC (Reserpine)
2.65e7
10.58
MRM of 3 Channels ES+
TIC (Salbutamol)
2.63e7
2.28
Time
MRM of 3 Channels ES-
TIC (Salicin)
8.57e4
3.94
MRM of 2 Channels ES-
TIC (Salicylic acid)
1.66e4
8.44
MRM of 3 Channels ES+
TIC (Scopolamine)
3.59e7
5.31
MRM of 3 Channels ES-
TIC (Sennoside A)
8.68e4
7.55
MRM of 3 Channels ES-
TIC (Sennoside B)
8.23e4
7.01
MRM of 3 Channels ES+
TIC (Serotonin_5-HT)
5.07e5
0.83
1.43
100
%
0
-0.00
2.00
4.00
6.00
8.00
10.00
12.00
14.00
16.00
18.00
20.00

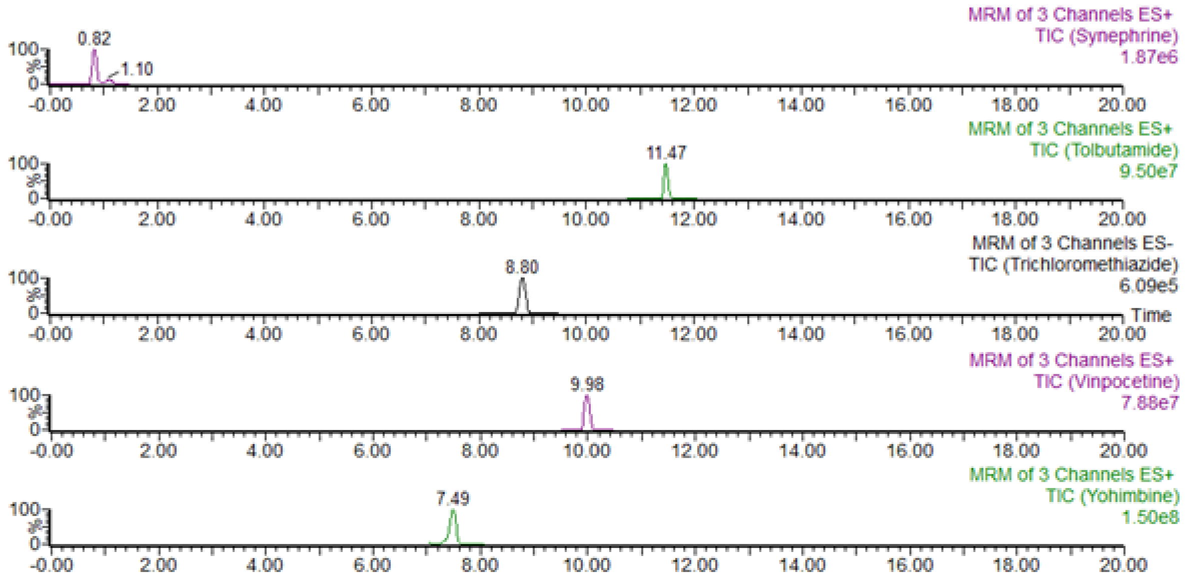

Figure S1. Chromatogram of 66 compounds at 0.05 mg L^{-1}

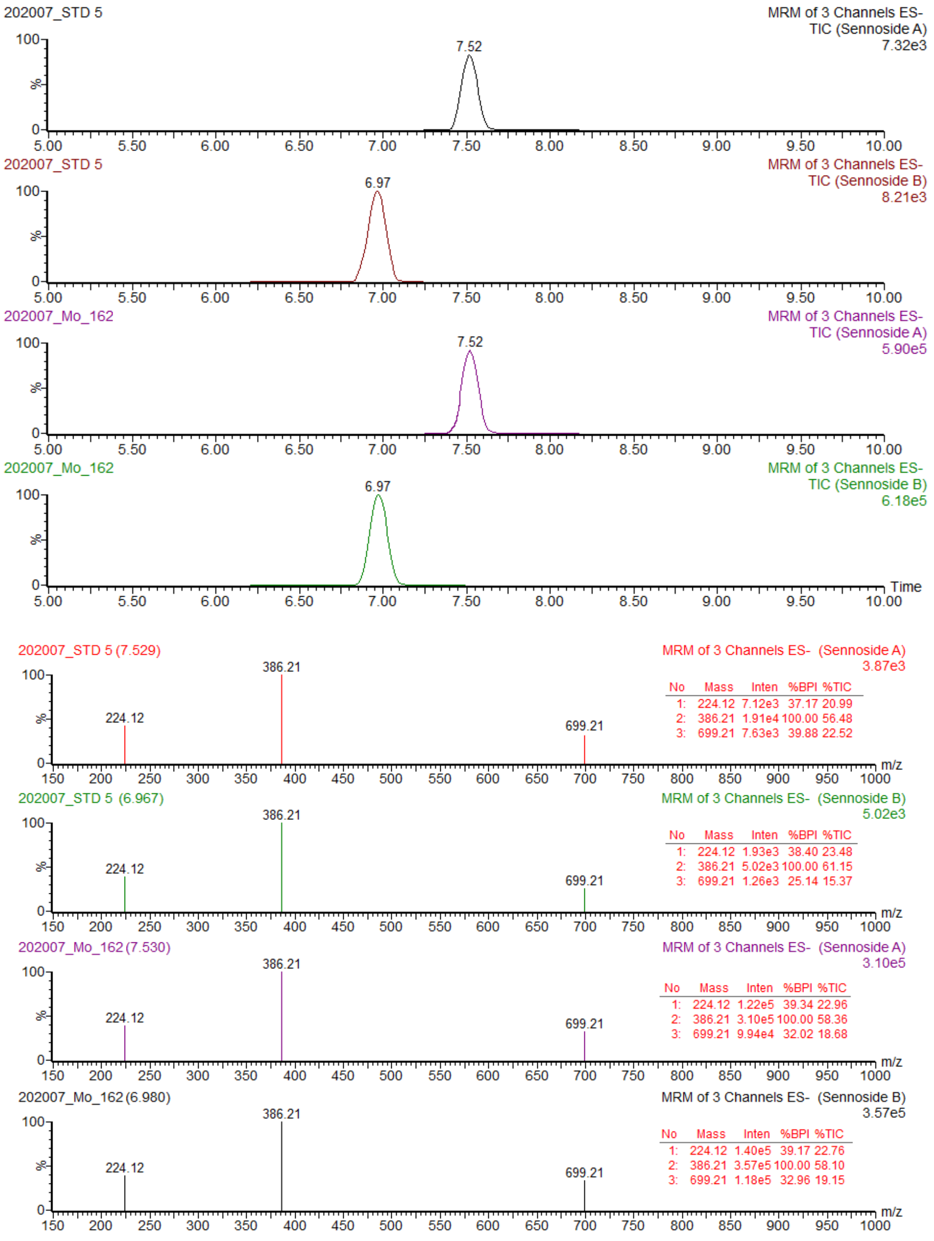

Figure S2. Example of chromatogram and mass spectra in sample (S-162, sennosides)

Table S1. Selection criteria of 66 target compounds based on Korean legislation.

Compounds	Prohibited compounds[1)]	Pharmaceutical	Not authorized ingredients[2)]	Compounds	Prohibited compounds[1)]	Pharmaceutical	Not authorized ingredients[2)]
2,4-Dinitrophenol (2,4-DNP)		O		Liothyronine	O	O	
7-keto-dehydroepiandrosterone (7-keto-DHEA)		O		Lorcaserine		O	
Amphetamine		O		Lovastatin		O	
Asarone			O	Magnoflorine			O
Atropine		O		Melatonin		O	
Berberine			O	Metformin		O	
beta-methylphenethylamine (BMPEA)		O		Methylclothiazide		O	
Bisacodyl		O		Mexamine (5-methoxytryptamine)		O	
Buformin		O		N-nitrosofenfluramine	O	O	
Cascaroside A	O		O	Noopept		O	
Cascaroside B	O		O	Oxilofrine		O	
Cascaroside C	O		O	Oxindole			O
Cascaroside D	O		O	Oxitriptan (5-hydroxytryptohpane)		O	
Chlorothiazide		O		Phendimetrazine		O	
Cimifugin			O	Phenformin		O	
Dehydroepiandrosterone (DHEA)		O		Phenolphtalein	O	O	
Echinacoside			O	Phentermine		O	
Ephedrine	O	O		Picamilon		O	
Fenfluramine	O	O		Rauwolscine (α-yohimbine)	O		O
Fluoxetine	O	O		Reserpine		O	
Glibenclamide	O	O		Salbutamol		O	
Gliclazide	O	O		Salicin			O
Glimepiride	O	O		Salicylic acid			O
Glipizide	O	O		Scopolamine		O	
Hesperidin			O	Sennoside A	O		O
Hydrastine			O	Sennoside B	O		O
Hydrochlorothiazide		O		Serotonin (5-hydroxytryptamine)		O	
Hydroflumethiazide		O		Synephrine		O	
Icariin	O		O	Tolbutamide		O	
Icaritin		O		Trichloromethiazide		O	
Kavain			O	Trigonelline			O
Levodopa		O		Vinpocetine			O
Levothyroxine	O	O		Yohimbine (β-yohimbine)	O		O

1) Prohibited by Food and Sanitation Act in Ministry of Food and Drug Safety in Korea
2) Not authorized food ingredients by Food Code in Korea

Table S2. The manufacturer and purity on target compounds

Compounds	Cas No.	Company	Purity (%)	Compounds	Cas No.	Company	Purity (%)
2,4-Dinitrophenol (2,4-DNP)	51-28-5	Sigma-aldrich	98.0	Liothyronine	6893-02-03	Sigma-aldrich	95.0
7-keto-dehydroepiandrosterone (7-keto-DHEA)	566-19-8	MFDS	99.9	Lorcaserine	616202-92-7	MFDS	99.0
Amphetamine	300-62-9	MFDS	99.9	Lovastatin	75330-75-5	USP	100
Asarone	2883-98-9	Sigma-aldrich	98.0	Magnoflorine	2141-09-05	Sigma-aldrich	98.0
Atropine	51-55-8	MFDS	97.0	Melatonin	73-31-4	Sigma-aldrich	98.0
Berberine	633-65-8	Sigma-aldrich	95.0	Metformin	657-24-9	Sigma-aldrich	98.0
beta-methylphenethylamine (BMPEA)	582-22-9	MFDS	95.0	Methylclothiazide	135-07-9	USP	99.0
Bisacodyl	603-50-9	Sigma-aldrich	98.0	Mexamine (5-methoxytryptamine1)	608-07-1	Sigma-aldrich	97.0
Buformin	692-13-7	Sigma-aldrich	98.0	N-nitrosofenfluramine	458-24-2	MFDS	99.0
Cascaroside A	53823-08-8	MFDS	98.0	Noopept	157115-85-0	Sigma-aldrich	99.7
Cascaroside B	53861-34-0	MFDS	98.0	Oxilofrine	365-26-4	TRC	96.0
Cascaroside C	53823-09-9	MFDS	97.0	Oxindole	59-48-3	Sigma-aldrich	97.0
Cascaroside D	53861-35-1	MFDS	99.0	Oxitriptan (5-hydroxytryptohpane)	4350-09-08	TRC	96.0
Chlorothiazide	58-94-6	Sigma-aldrich	98.0	Phendimetrazine	634-03-7	MFDS	99.9
Cimifugin	53-43-0	MFDS	99.0	Phenformin	834-28-6	Sigma-aldrich	100
Dehydroepiandrosterone (DHEA)	82854-37-3	Sigma-aldrich	99.0	Phenolphtalein	1977-09-08	Sigma-aldrich	98.0
Echinacoside	299-42-3	USP	90.0	Phentermine	122-09-8	MFDS	99.0
Ephedrine	458-24-2	Sigma-aldrich	99.0	Picamilon	34562-97-5	Sigma-aldrich	98.0
Fenfluramine	54910-89-3	MFDS	99.2	Rauwolscine (α-yohimbine)	6211-32-1	Sigma-aldrich	98.0
Fluoxetine	10238-21-8	MFDS	98.0	Reserpine	50-55-5	USP	99.0
Glibenclamide	21187-98-4	Sigma-aldrich	99.0	Salbutamol	18559-94-9	Sigma-aldrich	98.0
Gliclazide	93479-97-1	Sigma-aldrich	98.0	Salicin	138-52-3	MFDS	99.0
Glimepiride	29094-61-9	Sigma-aldrich	99.5	Salicylic acid	69-72-7	MFDS	99.9
Glipizide	520-26-3	Sigma-aldrich	99.8	Scopolamine	55-16-3	Sigma-aldrich	99.0
Hesperidin	118-08-1	Sigma-aldrich	98.0	Sennoside A	81-27-6	Wako	94.5
Hydrastine	58-93-5	USP	100	Sennoside B	128-57-4	Wako	94.5
Hydrochlorothiazide	135-09-1	USP	99.7	Serotonin (5-hydroxytryptamine)	50-67-9	Sigma-aldrich	98.5
Hydroflumethiazide	489-32-7	USP	98.0	Synephrine	1994-07-05	MFDS	99.0
Icariin	118525-40-9	Sigma-aldrich	99.0	Tolbutamide	64-77-7	Sigma-aldrich	98.0
Icaritin	3155-48-4	MFDS	99.0	Trichloromethiazide	133-67-5	USP	99.5
Kavain	59-92-7	Sigma-aldrich	95.0	Trigonelline	6138-41-6	Sigma-aldrich	100
Levodopa	51-48-9	Sigma-aldrich	100	Vinpocetine	42971-09-5	Sigma-aldrich	100
Levothyroxine	51-28-5	Sigma-aldrich	98.0	Yohimbine (β-yohimbine)	146-48-5	Sigma-aldrich	98.0

1) MFDS, ministry of food and drug safety; USP, U.S. pharmacopeia ;TRC, Toronto research chemicals

Table S3. The collected sample numbers based on sample type and product category

Sample type	Sexual performance enhancement	Weight loss	Muscular strengthening	Relaxation	Others	Total
Capsule	19	53	10	8	12	102
Tablet	5	8	2	2	4	21
Soft-gel	3	4	-	-	5	12
Powder	4	23	21	-	2	50
Tea bag	-	1	-	-	-	1
Energy Bar	-	2	-	-	-	2
Liquid	-	3	5	1	2	11
Product category						
Dietary supplements	28	92	38	10	25	194
Herbal supplements	3	1	-	1	-	5
Food supplements	-	1	-	-	1	2
Total	31	94	38	11	26	200
